# Development and external validation of an interpretable machine learning model for obesity-depression comorbidity in Korean and US adults

**DOI:** 10.3389/ijph.2026.1609153

**Published:** 2026-05-28

**Authors:** Yuwen Shangguan, Zhenhao Lin, Young-Je Sim, Kunpeng Wu, Yu Chu, Kunyi Huang, Fangxi Chen, Kangkang Ji, Fang Chen, Shangrui Liu

**Affiliations:** 1 Department of Exercise Physiology, Kunsan National University, Gunsan, Republic of Korea; 2 Yancheng Key Laboratory of Molecular Epigenetics, Yancheng Medical Research Center of Nanjing University Medical School, The First People’s Hospital of Yancheng, Yancheng, China; 3 Department of Health and Physical Education, The Education University of Hong Kong, Tai Po, Hong Kong SAR, China; 4 Department of Clinical Medical Research, Binhai County People’s Hospital, Binhai Clinical College, Yangzhou University Medical College, Yancheng, Jiangsu, China; 5 Department of Physical Education, Kyungpook National University, Daegu, Republic of Korea

**Keywords:** KNHANES, machine learning prediction, obesity-depression comorbidity, physical inactivity, SHAP interpretability

## Abstract

**Objective:**

To investigate the association between physical inactivity and obesity–depression comorbidity (ODC), defined as the co-occurrence of obesity and depression, and to develop an effective screening tool for identifying high-risk individuals to facilitate early intervention.

**Methods:**

Data were obtained from 3,357 physically inactive adults enrolled in the Korea National Health and Nutrition Examination Survey (KNHANES, 2007–2012). An XGBoost machine learning framework was applied to develop predictive models. Feature selection was conducted using random forest, and the prediction mechanism was interpreted with SHAP values. The model was validated internally using KNHANES 2011–2012 data and externally with the U.S. NHANES dataset.

**Results:**

The XGBoost model demonstrated good discriminative performance in internal validation (AUC = 0.783 and 0.744) and achieved an external validation AUC of 0.886. Feature importance analysis revealed that insulin concentration, white blood cell count, and height were the primary predictors of ODC, with insulin exerting the strongest influence.

**Conclusion:**

This study developed a high-performing and interpretable prediction model for ODC risk. SHAP-based interpretation identified insulin as the most influential predictor within the model, suggesting that metabolic factors may be important for ODC risk stratification.

## Introduction

The co-occurrence of obesity and depression, termed obesity-depression comorbidity (ODC), has emerged as a significant global public health challenge receiving heightened attention within healthcare systems and society [1–3]. Obesity represents a chronic, multifactorial disease state intricately associated with metabolic dysregulation, cardiovascular pathology, and mental health disturbances [4, 5]. Concurrently, depression—among the most prevalent affective disorders—exhibits strong bidirectional relationships with chronic somatic conditions, particularly obesity [6, 7]. Against the backdrop of evolving socioeconomic structures and lifestyle patterns, obesity and depression prevalence continues to escalate within adult populations globally. Their comorbid presentation has demonstrated increasing frequency, substantially impairing patients’ quality of life while imposing growing economic burdens on healthcare infrastructures and society [8, 9]. Although epidemiological associations between depression and obesity are well-documented, the underlying mechanistic pathways and causal sequences remain inadequately characterized [10]. The parallel increase in both conditions necessitates urgent development of interventions targeting their complex interplay. In the present study, ODC was treated as a concurrent comorbidity outcome rather than a directional transition from obesity to depression or from depression to obesity.

Current therapeutic modalities primarily encompass pharmacological and psychological approaches, yet these strategies frequently encounter limitations regarding sustained efficacy and treatment adherence [11, 12]. Given the multifactorial etiology of depression and obesity—involving genetic, environmental, and psychosocial determinants—innovative therapeutic paradigms are required to enhance outcomes and establish personalized intervention pathways [13, 14]. The identification and mechanistic dissection of pivotal factors in ODC development are consequently imperative for optimizing interventional efficacy. Previous research has established physical inactivity as a shared risk factor for both obesity and depression [15, 16], suggesting that augmented physical activity may complement conventional treatment strategies. Nevertheless, despite well-characterized associations between exercise engagement and these disease states, substantial knowledge gaps persist concerning their complex interactions, particularly within large-scale multi-cycle population-based survey data. Prior cross-sectional analyses have inadequately explored synergistic effects among physical activity, socioeconomic status, and dietary patterns, lacking systematic examination of comorbidity mechanisms. Contemporary machine learning methodologies and explainable artificial intelligence algorithms such as SHAP (Shapley Additive Explanations) have demonstrated considerable potential in health risk assessment and disease mechanism research through their capacity to model intricate relationships while enhancing interpretability [17, 18].

Given these research gaps—especially regarding multifactorial interactions and cross-sectional characteristics collected across multiple survey cycles—this study focuses on physically inactive adults. We employ an integrated machine learning and SHAP analytical framework to develop an interpretable risk prediction model for ODC and to quantify the relative contributions of key predictors at the model level. The model was specifically developed for risk stratification among physically inactive adults, a subgroup considered to be at elevated risk for obesity-depression comorbidity. Leveraging data from KNHANES (2007–2012), we examined how physical inactivity is associated with the co-occurrence of obesity and depression. Through integration of multidimensional data (demographic, behavioral, socioeconomic, clinical, and nutritional domains), this study identifies major predictors of ODC within the model and quantifies their relative contributions, thereby providing a basis for future risk stratification and hypothesis generation. Our principal objective was to address knowledge gaps in nonlinear pattern analysis and quantitative feature attribution via integration of machine learning and SHAP, thereby improving understanding of ODC-related risk patterns.

## Methods

### Data source and study population

Analytical data originated from the 2007–2012 Korea National Health and Nutrition Examination Survey (KNHANES) database administered by the Korea Centers for Disease Control and Prevention (CDC) [19, 20]. KNHANES constitutes a nationally representative continuous cross-sectional survey employing multistage stratified cluster sampling methodology. The survey comprises three distinct modules: health examinations, nutrition assessments, and health interviews. The institutional review board of the KCDC granted ethical approval for the study protocol, with all participants providing written informed consent.

**FIGURE 1:**
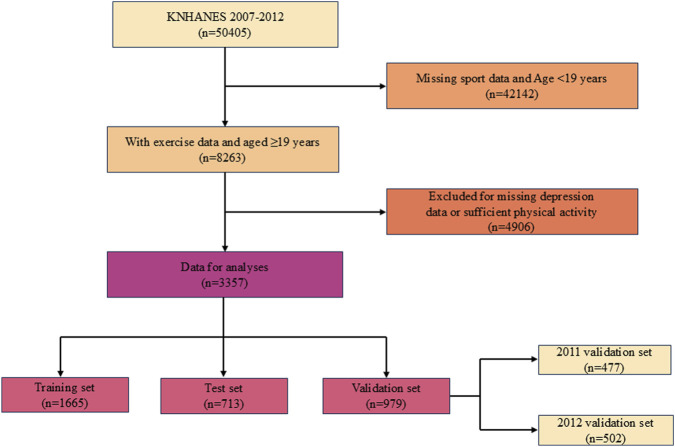
Population screening process (South Korea, 2007–2012).

The initial analytical cohort included 50,405 KNHANES participants (2007–2012). Screening procedures first excluded individuals with incomplete physical activity documentation, yielding 8,263 eligible subjects. Subsequent exclusions comprised participants meeting adequate physical activity thresholds (defined in Section *Definition of physically inactive population*) and those aged <19 years, leaving 3,750 individuals. Following exclusion of 393 subjects with missing depression-related metrics, the final analytical sample encompassed 3,357 adults. To assess temporal robustness within the same survey framework, the 2011 (n = 477) and 2012 (n = 502) KNHANES subsets were reserved as temporally separated internal validation cohorts. The remaining participants from 2007 to 2010 (n = 2,378) were randomly partitioned into training (n = 1,665) and testing (n = 713) subsets at a 7:3 ratio. Figure 1 presents the comprehensive participant selection workflow.

### Definition of physically inactive population

Consistent with World Health Organization (WHO) physical activity guidelines, this study evaluated individual activity levels using metabolic equivalent minutes per week (MET-min/week). Exclusion criteria incorporated missing essential data elements including weekly frequency and average daily duration of walking, moderate-intensity exercise, and vigorous-intensity exercise. Referencing WHO recommendations, minimum effective session durations were established: ≥15 min for vigorous exercise and ≥10 min for moderate exercise or walking. To minimize extreme value influence on cumulative activity calculations, session durations underwent Winsorization at the 99th percentile. Following KNHANES methodology, total weekly physical activity (PA) was computed as PA (MET-min/week) = MET coefficient × session duration × weekly frequency [21]. Based on WHO standards, participants achieving PA < 600 MET-min/week were classified as “physically inactive,” whereas those attaining PA ≥ 600 MET-min/week were designated “physically active” [21–23].

### Definition of obesity-depression comorbidity

This investigation employed Asian-specific diagnostic criteria for obesity classification and the Patient Health Questionnaire-9 (PHQ-9) for depression assessment to define obesity-depression comorbidity (ODC). Obesity categorization integrated body mass index (BMI) and waist circumference (WC) measurements: generalized obesity was defined as BMI ≥25 kg/m^2^; abdominal obesity was defined as WC ≥ 90 cm for males or ≥85 cm for females. Participants were consequently stratified into four mutually exclusive categories: 1) Non-obese (below threshold values for both BMI and WC); 2) Isolated abdominal obesity (WC exceeding threshold with subthreshold BMI); 3) Isolated generalized obesity (BMI exceeding threshold with subthreshold WC); and 4) Compound obesity (exceeding thresholds for both indices). For analytical purposes, categories 2–4 were collectively classified as obese. Depression status was assessed using the Patient Health Questionnaire-9 (PHQ-9; total score range: 0–27) [24]. Consistent with prior validation studies, a PHQ-9 score of ≥10 was used to indicate clinically significant depressive symptoms [25]. Thus, in this study, depression was operationally defined on the basis of symptom screening rather than physician-diagnosed depression.

### Candidate predictor variables

Based on existing literature and clinical expertise, this study incorporated multiple classes of potential predictor variables relevant to depression-obesity comorbidity, comprising: Demographic and sociological characteristics (sex, age, household income, educational attainment, marital status); health status indicators and disease history (hypertension, dyslipidemia, stroke, myocardial infarction, arthritis, diabetes, smoking status, alcohol consumption); clinical signs and laboratory parameters (systolic blood pressure, diastolic blood pressure, height, fasting glucose, insulin, total cholesterol, high-density lipoprotein cholesterol (HDL-C), triglycerides, hematocrit, ferritin, serum creatinine, vitamin D, white blood cell count, red blood cell count, platelet count); dietary intake metrics derived from 24-h recall (total food mass, total energy intake, water consumption, protein, fat, carbohydrates, calcium, phosphorus, iron, sodium, potassium, vitamin A, β-carotene, retinol, thiamine, riboflavin, niacin, vitamin C).

### Data preprocessing and machine learning modeling

The initial dataset contained 46 predictor variables (12 categorical, 34 continuous). To develop robust, generalizable prediction models, systematic data preprocessing and modeling procedures were implemented. Samples with missing values were excluded in a complete-case analysis, rather than being imputed, to avoid introducing additional model-based uncertainty across heterogeneous demographic, clinical, and nutritional variables during preprocessing. Subsequently, three feature selection methods—logistic regression, LASSO regression, and random forest—were applied to identify optimal predictive feature subsets. The LASSO method was selected for final feature subset construction based on five-fold cross-validated area under the receiver operating characteristic curve (AUC-ROC) performance in the training set. SHAP values were used only after final model development to interpret variable contributions within the XGBoost model; therefore, SHAP-based importance rankings were not intended to replicate the feature selection results obtained in the preprocessing stage. Pearson correlation coefficients were computed to address multicollinearity, retaining variables with stronger outcome associations when pairwise correlations exceeded 0.8. To address class imbalance, the Synthetic Minority Over-sampling Technique (SMOTE) was applied exclusively to the training set to improve recognition of the minority ODC-positive class [26]. No oversampling was performed in the internal test or external validation datasets, thereby reducing the risk of information leakage and overly optimistic performance estimates.

Multiple candidate models were trained and compared using SMOTE-processed data, including logistic regression, random forest, XGBoost, decision tree, naïve Bayes, K-nearest neighbors, and radial basis function (RBF) kernel support vector machines (SVM). Hyperparameters were systematically optimized via grid search with five-fold cross-validation on the internal test set. Following comprehensive performance comparisons, the optimally parameterized XGBoost model was selected for final evaluation on temporally independent external validation sets (2011 and 2012 data) to assess clinical generalizability and stability. Crucially, SMOTE application was restricted to training data construction, while test and external validation sets retained original class distributions to prevent information leakage and ensure objective evaluation.

### External validation strategy

To objectively evaluate model generalizability, the 2005–2020 U.S. National Health and Nutrition Examination Survey (NHANES) cohort served as an independent external validation dataset. Applying identical inclusion/exclusion criteria as KNHANES produced a validation cohort of 2,070 participants (demographic characteristics in Supplementary Table S1). NHANES was selected as an accessible and well-characterized independent population-based dataset for external testing; however, it was not intended to represent the most culturally or clinically comparable population to South Korea. We did not perform U.S.-specific recalibration. Instead, the NHANES analysis was intended to assess the external discrimination and transportability of the KNHANES-derived model in an independent population setting. In the external validation cohort, obesity was defined according to U.S.-appropriate criteria to preserve the clinical relevance of outcome ascertainment in that population. Within this cohort, XGBoost performance was benchmarked against conventional algorithms including logistic regression, SVM, and random forest, primarily using area under the receiver operating characteristic curve (AUC) for discriminative ability assessment. To interpret the optimal model’s (XGBoost) prediction patterns and identify influential predictors, mean absolute SHAP values were computed across the validation cohort, ensuring interpretative consistency and local explanation accuracy.

### Statistical analysis

Binary prediction performance for ODC was comprehensively evaluated through systematic comparison of multiple machine learning algorithms: XGBoost, decision tree, logistic regression, naïve Bayes, K-nearest neighbors, random forest, and RBF-kernel SVM. Performance was assessed multidimensionally: Fundamental metrics included error rate and accuracy; class imbalance was addressed via Fβ-score (integrating precision and recall); discriminatory capacity was measured by AUC, sensitivity, and specificity; precision-recall balance was evaluated via precision-recall AUC (PR AUC). Calibration curves assessed agreement between predicted probabilities and observed event rates. Decision curve analysis (DCA) quantified clinical utility by comparing net benefits across decision thresholds. Following comparative evaluation using these metrics, the best-performing XGBoost model underwent final validation and interpretability analysis. SHAP (SHapley Additive exPlanations) values enabled quantification of individualized feature contributions to ODC predictions, enhancing model interpretability. Finally, an interactive online risk prediction tool was developed using the R Shiny framework based on the validated XGBoost architecture. All analyses were conducted in R (version 4.4.1) employing critical packages: DMwR, ggcor, mlr3, mlr3benchmark, mlr3extralearners, kernelshap, and shapviz. Two-sided statistical tests were applied with significance defined as p < 0.05.

## Results

### Baseline characteristics of the study population

The final analytical cohort comprised 3,357 physically inactive adult participants, stratified into training (n = 1,665), testing (n = 713), and two temporally independent internal validation cohorts (2011: n = 477; 2012: n = 502). Baseline characteristic analysis revealed no statistically significant differences in core demographic and clinical variables—including gender distribution, household income, educational attainment, marital status, and chronic disease history—across datasets (p > 0.05), with all standardized mean differences (SMD) below the 0.1 threshold. Though statistically significant variations existed for select metabolic and nutritional indicators (p < 0.05), their SMD values remained below the 0.3 benchmark for clinical relevance. Compared to the training set, validation cohorts exhibited elevated high-density lipoprotein cholesterol (HDL-C) and hematocrit concentrations, while demonstrating significantly reduced vitamin D levels and diminished dietary intakes of carbohydrates, phosphorus, sodium, and potassium (all p < 0.01; SMDs < 0.227). Statistically significant but clinically marginal differences were also observed for diastolic blood pressure, height, insulin concentrations, and alcohol consumption patterns (p < 0.05; SMDs<0.106). With all variables exhibiting SMDs below 0.3, the datasets demonstrated satisfactory clinical comparability for subsequent machine learning modeling and validation procedures (complete data in Supplementary Table S2).

**FIGURE 2:**
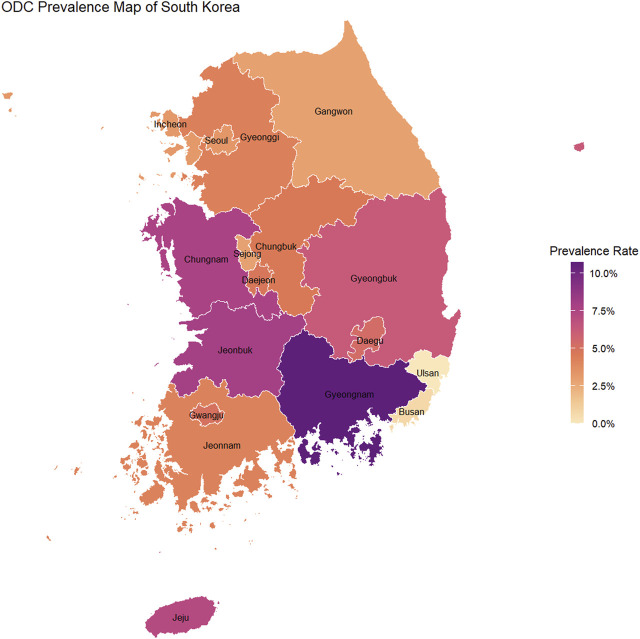
Geographical distribution heatmap of depression-obesity comorbidity prevalence among Korean adults (South Korea, 2007–2012).

To elucidate geographical heterogeneity in obesity-depression comorbidity (ODC) burden, we generated a spatial heatmap depicting ODC prevalence probabilities across Korean administrative regions (Figure 2). Results revealed marked epidemiological disparities: South Gyeongsang Province exhibited the highest prevalence (10.7%), followed sequentially by South Chungcheong, North Jeolla, Jeju Island, and North Gyeongsang (all >6.0%). Remaining regions demonstrated relatively uniform distribution patterns. This geographical stratification establishes critical context for subsequent subgroup analyses and informs assessments of model generalizability across diverse populations.

### Feature selection

**FIGURE 3:**
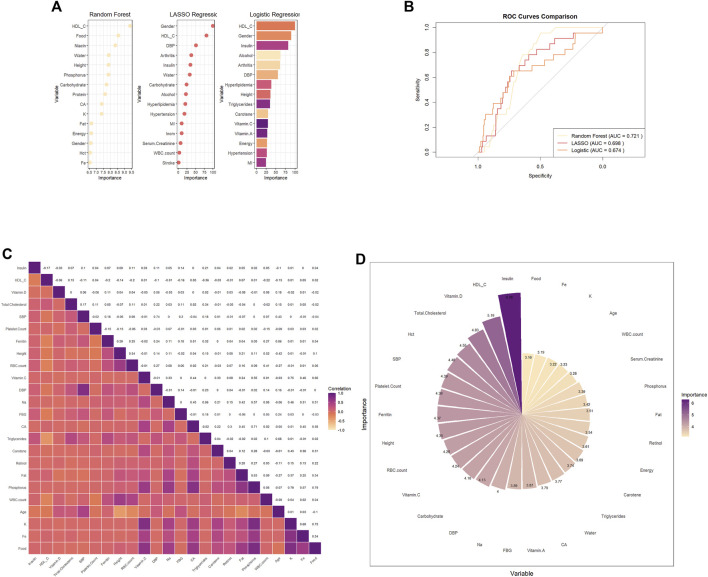
Feature selection and preliminary comparison of model performance (South Korea, 2007–2012). **(A)** Comparison of feature importance rankings generated by logistic regression, least absolute shrinkage and selection operator regression, and random forest. **(B)** Receiver operating characteristic curves of the three models in the training dataset. **(C)** Correlation matrix after removal of collinear features. **(D)** Contribution scores of the 30 most important predictors identified by random forest.

To construct an optimal predictive feature subset, we systematically evaluated three feature selection methodologies—logistic regression, LASSO regression, and random forest—on the training cohort (Figure 3A). Comparative assessment using five-fold cross-validated area under the receiver operating characteristic curve (AUC-ROC) demonstrated the random forest approach achieving superior discriminatory capacity (AUC = 0.721), significantly outperforming LASSO regression (AUC = 0.698) and logistic regression (AUC = 0.674) (Figure 3B). Consequently, random forest was selected as the definitive feature selection technique. Variable importance ranking yielded the top 30 predictors (Figure 3D), encompassing metabolic biomarkers, hematological indices, nutritional parameters, and demographic characteristics. To mitigate multicollinearity effects, Pearson correlation matrices were computed (Supplementary Figure S1). For feature pairs exhibiting correlation coefficients >0.8, we retained variables demonstrating stronger associations with the outcome (ODC), excluding six redundant parameters: serum creatinine, vitamin A, water intake, carbohydrate intake, total energy intake, and hematocrit. This refinement process yielded a final feature set comprising 24 core variables for model construction (Figure 3C). These results reflect the performance of alternative feature selection strategies when all candidate variables were initially entered for screening, with the aim of identifying the most suitable method for constructing an optimal predictor subset. Thus, the logistic regression result shown in Figure 3 represents a preliminary feature selection performance rather than the final performance of a logistic regression classifier.

### Model performance comparison and optimization

**FIGURE 4:**
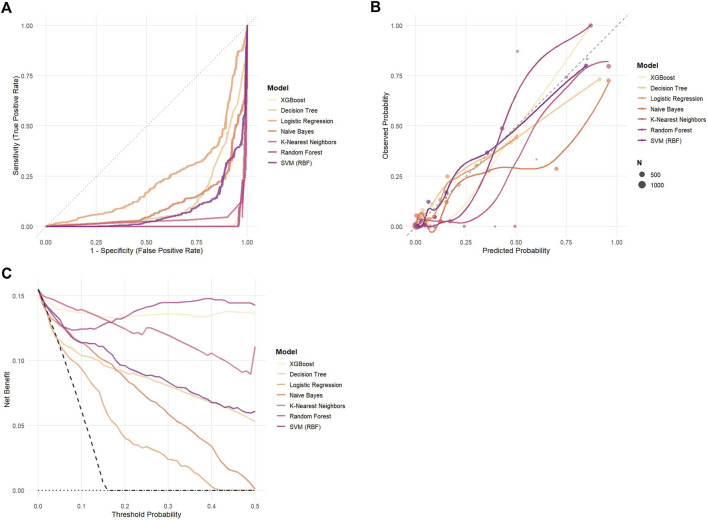
Model validation results after synthetic minority oversampling technique resampling (South Korea, 2007–2012). **(A)** Receiver operating characteristic curves. **(B)** Decision curve analysis. **(C)** Calibration curves.

After the optimal feature selection method was determined and the retained variables were used to construct the final dataset, seven machine learning classifiers were compared to identify the best-performing predictive model. Comprehensive algorithm comparison (Supplementary Figure S2; Table 1) revealed that XGBoost and random forest models substantially outperformed alternatives. Both attained accuracy exceeding 98%, ROC AUC surpassing 0.98, sensitivity approaching 100%, specificity exceeding 92%, and precision-recall AUC (PR AUC) above 0.97 when evaluated on the SMOTE-processed training data (Figure 4). Their respective Brier scores—0.0240 (ranked first) and 0.0244 (ranked second)—indicated optimal probability calibration. Moderate performance was observed for K-nearest neighbors and support vector machines, while decision trees, naive Bayes, and logistic regression showed markedly inferior metrics (notably Brier scores >0.14, specificity <75%, and PR AUC <0.5), suggesting inadequate predictive stability (detailed metrics in Supplementary Table S3). On the independent test set preserving original class distribution, the XGBoost model maintained strong generalization capability (Supplementary Table S4), achieving an AUC of 0.750—confirming robust discriminatory power. Following clinical sensitivity optimization using a 0.1 decision threshold, the model attained a recall rate of 65.38% (95% CI: 54.2%–75.4%), though precision remained constrained at 8.46%, reflecting inherent sensitivity-precision trade-offs in severely imbalanced data (F1 score = 0.150; accuracy = 72.93%). However, this sensitivity-oriented threshold was associated with a low positive predictive value, indicating a substantial false-positive burden and limiting the model’s suitability as a standalone clinical screening tool. The test set confusion matrix further delineated classification performance of the optimized XGBoost model (Supplementary Table S5).

**TABLE 1:** Performance evaluation results of seven machine learning models (South Korea, 2007–2012).

Model	Error Rate	Accuracy	F-beta	ROC AUC	Sensitivity	Specificity	PR AUC
Random forest	0.0122	0.9878	0.9928	0.9895	1.0000	0.9212	0.9783
XGBoost	0.0186	0.9814	0.9890	0.9883	0.9887	0.9418	0.9715
K-nearest neighbors	0.0441	0.9559	0.9734	0.9507	0.9523	0.9760	0.7624
SVM (RBF)	0.0939	0.9061	0.9459	0.9184	0.9711	0.5514	0.7617
Decision tree	0.1019	0.8981	0.9400	0.8640	0.9447	0.6438	0.5958
Naive Bayes	0.1539	0.8461	0.9063	0.8864	0.8807	0.6575	0.5950
Logistic regression	0.1576	0.8424	0.9126	0.7661	0.9742	0.1233	0.3469

Abbreviations: XGBoost, eXtreme Gradient Boosting; SVM (RBF), Support Vector Machine (Radial Basis Function); F-beta, harmonic mean of precision and recall with adjustable weighting toward recall; ROC AUC, area under the receiver operating characteristic curve; PR AUC, area under the precision-recall curve.

Systematic evaluation of seven machine learning algorithms (Supplementary Figure S2) employed SMOTE-oversampled training data (ODC positive:negative ratio = 1:5). Through grid search coupled with five-fold cross-validation for hyperparameter optimization, the optimal configuration was determined (learning rate eta = 0.1, max_depth = 6, subsample = 0.8, lambda = 1.0), with early stopping regularization controlling overfitting. As documented in Supplementary Table S6, peak performance occurred at the 44th iteration (test set AUC = 0.750), establishing XGBoost as the definitive predictive framework.

### Internal and external model validation

To rigorously assess generalizability and clinical utility, the XGBoost model underwent comprehensive validation using two temporally distinct internal cohorts (2011: n = 477; 2012: n = 502). As presented in Supplementary Table S7, the model demonstrated excellent temporal discriminative capability (Supplementary Figure S3): ROC curve analysis yielded AUC values of 0.783 (95% CI: 0.702–0.864) for the 2011 cohort and 0.744 (95% CI: 0.652–0.835) for the 2012 cohort. Implementation of a low decision threshold (0.1) optimized for screening sensitivity achieved detection rates of 84.2% (95% CI: 73.1%–91.4%) and 70.0% (95% CI: 55.9%–81.2%) in the respective cohorts, successfully capturing over two-thirds of true positive cases—meeting fundamental requirements for early screening instruments.

In external validation using the U.S. NHANES cohort, the XGBoost model achieved an AUC of 0.886, allowing direct comparison with the internal validation performance reported above. The random forest classifier ranked second (AUC = 0.858), followed by radial basis function (RBF) kernel support vector machine (AUC = 0.831). Remaining models demonstrated comparatively limited predictive capacity: K-nearest neighbors (AUC = 0.795), logistic regression (AUC = 0.778), naive Bayes (AUC = 0.759), and decision tree (AUC = 0.667), See Supplementary Table S8 for details. Comparative ROC curves are depicted in Supplementary Figure S4.

### SHAP interpretability analysis

**FIGURE 5:**
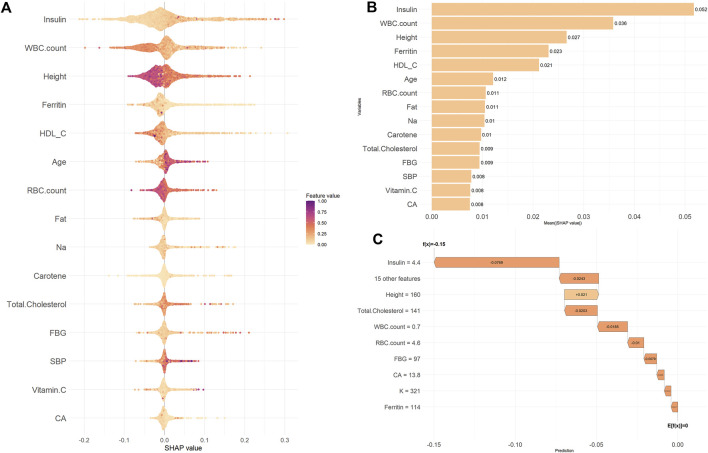
Model interpretability visualizations for the extreme gradient boosting model (South Korea, 2007–2012). **(A)** Shapley additive explanations beeswarm plot. **(B)** Shapley additive explanations global importance bar plot. **(C)** Shapley additive explanations waterfall plot.

SHAP (SHapley Additive exPlanations) was used to interpret the XGBoost model’s prediction patterns. Feature importance analysis (Figure 5) identified insulin concentration as the predominant predictor of obesity-depression comorbidity (mean |SHAP| = 0.052), exerting substantially greater influence than secondary contributors: white blood cell count (0.036), height (0.027), ferritin (0.023), HDL-C (0.021), and age (0.012). SHAP summary plots indicated that elevated insulin, advanced age, increased systolic blood pressure, and higher white blood cell counts were associated with higher predicted ODC probability, whereas greater height and elevated HDL-C concentrations were associated with lower predicted ODC probability. Supplementary Figure S5 provides additional SHAP dependence visualizations.

External validation SHAP analysis using the NHANES cohort further delineated the optimal model’s decision architecture and feature contribution patterns (Supplementary Figure S6). Insulin was reconfirmed as the most influential predictive variable, with its mean absolute SHAP value substantially exceeding those of other features—underscoring its centrality in model discrimination. Key predictors including age, retinol, height, and fasting glucose (fglu) followed in descending order of importance, exhibiting remarkable concordance with KNHANES-derived SHAP results. This cross-cohort reproducibility enhances model credibility and suggests that integrated metabolic, nutritional, and developmental features may provide a robust predictive foundation across populations.

### Online prediction tool demonstration

Based on the rigorously validated XGBoost framework, we developed a clinically oriented online prediction instrument (accessible at https://zhlapp.shinyapps.io/Korea_ODC-shap-model/). Implemented via the R Shiny platform, this tool provides interactive risk assessment functionality, enabling healthcare practitioners to input 24 core indicators—including insulin concentration, white blood cell count, height, and age—for real-time ODC risk stratification, as visually demonstrated in Supplementary Figure S7.

## Discussion

This investigation established an XGBoost-based machine learning framework for predicting obesity-depression comorbidity (ODC) risk among physically inactive adults, leveraging KNHANES data. SHAP methodology provided interpretable information on key predictors and their interactions within the model. Principal findings include: (1) The developed XGBoost model demonstrated robust discriminatory capacity in internal validation (2011 and 2012 KNHANES cohorts; AUCs = 0.783 and 0.744 respectively) and exceptional generalizability in an independent NHANES validation cohort (AUC = 0.886), significantly outperforming comparator models and confirming clinical utility for early ODC detection; (2) SHAP interpretability analysis identified insulin concentration as the predominant ODC predictor (highest mean absolute SHAP value), followed sequentially by white blood cell count, age, retinol, height, and fasting glucose—highlighting central roles of metabolic regulation, nutritional status, and developmental indicators [27, 28], however, given the cross-sectional design of the present study, these findings should be interpreted as associations with model prediction rather than evidence of temporal or causal pathways underlying ODC; (3) Marked geographical heterogeneity in ODC prevalence across South Korean regions (e.g., peak prevalence of 10.7% in South Gyeongsang) provides epidemiological foundations for targeted public health initiatives; (4) Integration of machine learning with SHAP methodology effectively quantified individualized contributions of multidimensional features (demographic, clinical, nutritional) to ODC risk and delineated their complex nonlinear association patterns.

SHAP analysis consistently identified insulin concentration as the most influential ODC predictor across both internal (KNHANES) and external (NHANES) validation cohorts. Insulin resistance—a core pathophysiological feature of obesity—has been mechanistically linked to depressive symptomatology in prior research [27–29]. Hyperinsulinemia and impaired insulin signaling may promote emotional dysregulation through disruptions in central neurotransmitter metabolism (e.g., dopamine), neuroplasticity, and hypothalamic-pituitary-adrenal (HPA) axis function [30–33]. By quantitatively establishing insulin’s centrality in ODC risk prediction through machine learning, this study highlights a potentially important association between metabolic dysregulation and metabolic-mental health comorbidity. The substantial contribution of white blood cell count (second-highest SHAP value) suggests that systemic low-grade inflammation may be associated with the co-occurrence of obesity and depression [34–36], though specific inflammatory biomarkers were not directly assayed. The positive association with advancing age may reflect cumulative effects of chronic disease burden, psychosocial stressors, or physiological decline [37, 38]. Conversely, the inverse associations observed for greater height and elevated HDL-C concentrations may reflect differences in growth-related exposures and cardiometabolic health status in relation to ODC. These predictors suggest that ODC is associated with complex multisystem patterns spanning metabolic, inflammatory, and developmental domains.

This study substantiates the superiority of machine learning algorithms, particularly XGBoost, in predicting complex outcomes like ODC that involve nonlinear interactions among demographic, behavioral, metabolic, and nutritional determinants [39–41]. Compared to conventional regression approaches, XGBoost more effectively captures intricate patterns and interaction effects within high-dimensional data, achieving superior discriminatory performance in both internal and external validations (AUC >0.74). Crucially, through integration of SHAP (SHapley Additive exPlanations)—an explainable artificial intelligence (XAI) technique—we successfully demystified the decision logic of this sophisticated model [42, 43]. SHAP values not only objectively quantified individualized predictor contributions to ODC risk (e.g., insulin’s dominant role) but dependence plots also visually revealed nonlinear relationships between key variables (e.g., insulin, age, systolic blood pressure, white blood cell count) and disease probability. This methodological synthesis enhances model transparency and clinical interpretability by helping translate risk scores into interpretable prediction patterns. The resultant online prediction tool (Supplementary Figure S7) may support clinical practice by helping identify high-risk individuals and informing further clinical assessment. In practice, clinicians could enter routinely available demographic, clinical, and laboratory variables into the web-based interface to obtain an individualized predicted risk of obesity-depression comorbidity. This output may be used to support preliminary risk stratification and to identify patients who may benefit from further psychological or metabolic assessment, rather than to establish a diagnosis independently.

This investigation presents the first geographical heatmap of ODC risk distribution across South Korea (Figure 2), revealing substantial regional heterogeneity (highest burden in South Gyeongsang). Such disparities may originate from inter-regional variations in socioeconomic status, healthcare access, cultural practices (e.g., dietary habits, physical activity norms), or environmental exposures. These findings provide critical epidemiological foundations for South Korea and comparable settings to develop regionalized precision prevention strategies. In high-prevalence regions (e.g., South Gyeongsang, South Chungcheong), community health initiatives should prioritize physical activity promotion, nutritional quality improvement, and enhanced access to integrated metabolic-mental health screening services.

### Study limitations

Several methodological constraints warrant acknowledgment: First, the development dataset was based on KNHANES 2007–2012, and temporal changes in lifestyle patterns, obesity prevalence, mental health awareness, and public health policies over the past decade may limit the model’s direct applicability to contemporary populations. Future studies should therefore assess the temporal transportability of the model using more recent datasets and update or recalibrate it as needed. Second, depression was defined using PHQ-9 screening rather than a structured clinical diagnosis. Third, missing data were handled using complete-case analysis without imputation. Although this approach avoided additional assumptions introduced by imputation models, it reduced the effective sample size and may have introduced selection bias if the missingness mechanism was not completely random. Future studies should evaluate the robustness of the findings using multiple imputation or other sensitivity analyses. Fourth, though demonstrating robust performance in the NHANES validation cohort, population-specific genetic backgrounds, cultural contexts, and social structures in South Korea may limit global generalizability, necessitating further validation across diverse populations, Future research should prioritize validation and recalibration in East Asian populations that are more comparable (such as Chinese or Japanese populations). Fifth, feature engineering excluded highly correlated variables (r > 0.8); while statistically justified, this process may have omitted biologically relevant indicators (e.g., vitamin A, hematocrit). Sixth, although the model achieved acceptable sensitivity at the optimized decision threshold of 0.1, its precision remained relatively low, indicating a substantial false-positive burden. In practical clinical settings, this may reduce efficiency, contribute to alert fatigue among clinicians, and lead to unnecessary follow-up assessments or anxiety in individuals incorrectly classified as high risk. Therefore, the model should be regarded as a preliminary risk stratification tool rather than a standalone screening or diagnostic instrument. Finally, although obesity in the NHANES cohort was defined using U.S.-appropriate criteria, the absence of U.S.-specific recalibration means that the external validation results should still be interpreted primarily in terms of discrimination and transportability rather than calibration equivalence.

### Conclusion

Utilizing a large-scale population-based dataset, this study developed and validated an interpretable XGBoost model for ODC risk prediction in physically inactive adults. SHAP analysis identified insulin as the most influential predictor within the model, indicating that metabolic variables may play an important role in model-based risk stratification. However, these feature-attribution results reflect predictive relevance within the algorithm rather than confirmed biological mechanisms. Accordingly, the findings should be interpreted as hypothesis-generating and supportive of further prospective, experimental, and interventional studies.

## Data Availability

The data used in this study are publicly available and can be freely downloaded from the KNHANES website (https://knhanes.kdca.go.kr/).
