# Supplementary material for: Development and external validation of an interpretable machine learning model for obesity-depression comorbidity in Korean and US adults
Source: Int J Public Health. 2026 May 28;71:1609153. doi: 10.3389/ijph.2026.1609153 (PMC13254829; doi:10.3389/ijph.2026.1609153)
Supplement: Supplementary file 1 [file Supplementaryfile1.docx]

**Supplementary material**

**Supplementary Table 1:** External Validation Cohort: NHANES Demographic and Clinical Characteristics

**Supplementary Table 2:** KNHANES baseline information.

**Supplementary Table 3:** Model Performance Ranking (Based on Brier Score)

**Supplementary Table 4:** Model Performance Metrics on the Independent Test Set

**Supplementary Table 5:** Confusion Matrix on the Test Set

**Supplementary Table 6:** Optimal Model Training Parameters

**Supplementary Table 7:** Performance Differences Between Validation Cohorts

**Supplementary Table 8:** External Validation of Model Performance Differences in NHANES

**Supplementary Figure 1:** Pearson association coefficient plot (prior to removing collinear variables)

**Supplementary Figure 2:** Ridge Plot Distribution of Performance Metrics for Seven Machine Learning Models

**Supplementary Figure 3:** ROC curve for the KNHANES internal validation set

**Supplementary Figure 4:** ROC curve for the NHANES external validation set

**Supplementary Figure 5:** SHAP Dependency Plot for KNHANES Data.

**Supplementary Figure 6:** ROC curve for the NHANES external validation set

**Supplementary Figure 7:** Real-time Visualization of Risk Probability Stratification Output from Online Prediction Tool

**Supplementary Table 1:** External Validation Cohort: NHANES Demographic and Clinical Characteristics

| Variable | Test set (n=620) | Training set (n=1449) | p-value | Test Type | SMD |
| --- | --- | --- | --- | --- | --- |
| Age, mean (SD) | 51.79 (16.87) | 51.20 (17.01) | 0.471 |  | 0.035 |
| Gender, n (%) |  |  | 0.309 | exact | 0.051 |
| Male | 256 (41.3) | 635 (43.8) |  |  |  |
| Female | 364 (58.7) | 814 (56.2) |  |  |  |
| Race, n (%) |  |  | 0.593 | exact | 0.082 |
| Non-Hispanic White | 299 (48.2) | 658 (45.4) |  |  |  |
| Non-Hispanic Black | 118 (19.0) | 305 (21.0) |  |  |  |
| Mexican American | 96 (15.5) | 217 (15.0) |  |  |  |
| Other Hispanic | 44 (7.1) | 124 (8.6) |  |  |  |
| Other Race | 63 (10.2) | 145 (10.0) |  |  |  |
| Marital Status, n (%) |  |  | 0.348 | exact | 0.071 |
| Married/Living with Partner | 399 (64.4) | 885 (61.1) |  |  |  |
| Widowed/Divorced/Separated | 134 (21.6) | 351 (24.2) |  |  |  |
| Never married | 87 (14.0) | 213 (14.7) |  |  |  |
| Education, n (%) |  |  | 0.355 | exact | 0.070 |
| Below high school | 43 (6.9) | 122 (8.4) |  |  |  |
| High school | 220 (35.5) | 535 (36.9) |  |  |  |
| Above high school | 357 (57.6) | 792 (54.7) |  |  |  |
| PIR, mean (SD) | 2.16 (0.79) | 2.14 (0.78) | 0.633 |  | 0.023 |
| Smoking, n (%) |  |  | 0.612 | exact | 0.048 |
| Never | 350 (56.5) | 812 (56.0) |  |  |  |
| Former | 165 (26.6) | 367 (25.3) |  |  |  |
| Now | 105 (16.9) | 270 (18.6) |  |  |  |
| Alcohol, n (%) |  |  | 0.591 | exact | 0.050 |
| Never | 79 (12.7) | 198 (13.7) |  |  |  |
| Former | 106 (17.1) | 268 (18.5) |  |  |  |
| Now | 435 (70.2) | 983 (67.8) |  |  |  |
| PA2, n (%) | 620 (100.0) | 1449 (100.0) | NA | exact | <0.001 |
| Height, mean (SD) | 166.91 (10.02) | 166.82 (9.82) | 0.853 |  | 0.009 |
| WAIST, mean (SD) | 98.71 (15.69) | 100.02 (16.72) | 0.098 |  | 0.081 |
| BMI, mean (SD) | 28.83 (6.32) | 29.48 (7.09) | 0.050 |  | 0.096 |
| SBP, mean (SD) | 69.44 (11.24) | 70.09 (11.93) | 0.245 |  | 0.056 |
| DBP, mean (SD) | 121.96 (17.17) | 123.97 (18.46) | 0.021 |  | 0.113 |
| PHQ9, mean (SD) | 0.07 (0.26) | 0.09 (0.28) | 0.236 |  | 0.058 |
| Hypertension, n (%) |  |  | 0.809 | exact | 0.014 |
| No | 344 (55.5) | 794 (54.8) |  |  |  |
| Yes | 276 (44.5) | 655 (45.2) |  |  |  |
| Diabetes, n (%) |  |  | 0.776 | exact | 0.052 |
| No | 434 (71.4) | 1002 (69.2) |  |  |  |
| Yes | 177 (28.5) | 447 (30.9) |  |  |  |
| Hyperlipidemia, n (%) |  |  | 0.782 | exact | 0.015 |
| No | 159 (25.6) | 362 (25.0) |  |  |  |
| Yes | 461 (74.4) | 1087 (75.0) |  |  |  |
| ML, n (%) |  |  | 0.542 | exact | 0.031 |
| No | 565 (91.1) | 1333 (92.0) |  |  |  |
| Yes | 55 (8.9) | 116 (8.0) |  |  |  |
| Stroke, n (%) |  |  | 0.475 | exact | 0.042 |
| No | 605 (97.6) | 1403 (96.9) |  |  |  |
| Yes | 15 (2.4) | 45 (3.1) |  |  |  |
| ODC, n (%) |  |  | 0.625 | exact | 0.027 |
| No | 583 (94.0) | 1353 (93.4) |  |  |  |
| Yes | 37 (6.0) | 96 (6.6) |  |  |  |
| X25.OH.D, mean (SD) | 62.88 (25.56) | 63.18 (26.73) | 0.808 |  | 0.012 |
| Fasting Glucose, mean (SD) | 5.95 (1.60) | 6.06 (2.03) | 0.245 |  | 0.058 |
| Fasting Insulin, median [IQR] | 9.41 [6.29, 15.53] | 9.74 [6.22, 15.89] | 0.640 | nonnorm | 0.019 |
| Triglycerides, median [IQR] | 103.00 [71.00, 151.25] | 106.00 [74.00, 155.00] | 0.319 | nonnorm | 0.018 |
| Total Cholesterol, mean (SD) | 192.15 (40.10) | 194.81 (41.79) | 0.179 |  | 0.065 |
| HDL-C, mean (SD) | 54.30 (15.48) | 54.20 (15.35) | 0.899 |  | 0.006 |
| RBC, mean (SD) | 4.71 (0.53) | 4.72 (0.49) | 0.468 |  | 0.034 |
| WBC, mean (SD) | 6.67 (1.97) | 6.80 (2.16) | 0.184 |  | 0.065 |
| Iron (mg), median [IQR] | 13.43 [10.06, 17.58] | 13.03 [9.77, 17.56] | 0.844 | nonnorm | 0.029 |
| Alpha-carotene, median [IQR] | 117.50 [32.50, 515.75] | 101.50 [30.50, 478.00] | 0.410 | nonnorm | 0.034 |
| Beta-carotene, median [IQR] | 1310.00 [495.00, 2896.25] | 1191.50 [451.50, 2759.00] | 0.078 | nonnorm | 0.093 |
| Carotene Retinol Equivalent, median [IQR] | 118.97 [43.43, 263.78] | 107.02 [39.60, 253.44] | 0.087 | nonnorm | 0.088 |
| Vitamin A, mean (SD) | 620.65 (544.83) | 589.93 (480.15) | 0.201 |  | 0.060 |
| Vitamin C, mean (SD) | 81.53 (69.25) | 82.43 (70.95) | 0.790 |  | 0.013 |
| Vitamin E, mean (SD) | 7.66 (4.39) | 7.37 (4.31) | 0.166 |  | 0.066 |
| Riboflavin, mean (SD) | 1.97 (0.88) | 1.98 (0.87) | 0.815 |  | 0.011 |
| Niacin, mean (SD) | 23.89 (10.86) | 23.50 (10.37) | 0.438 |  | 0.037 |
| Thiamin, mean (SD) | 1.52 (0.66) | 1.53 (0.67) | 0.741 |  | 0.016 |
| Calcium, mean (SD) | 856.51 (392.75) | 862.97 (407.16) | 0.738 |  | 0.016 |
| Magnesium, mean (SD) | 284.79 (113.66) | 278.52 (106.36) | 0.229 |  | 0.057 |
| Iron, mean (SD) | 14.18 (6.37) | 14.37 (6.81) | 0.548 |  | 0.029 |
| Dietary Intake |  |  |  |  |  |
| Carbohydrates, mean (SD) | 235.50 (86.70) | 242.52 (96.72) | 0.120 |  | 0.076 |
| Energy, mean (SD) | 1949.21 (681.85) | 1966.53 (720.37) | 0.611 |  | 0.025 |
| Total Fat, mean (SD) | 75.43 (34.57) | 74.55 (33.23) | 0.583 |  | 0.026 |
| Protein, mean (SD) | 77.80 (29.48) | 76.78 (29.61) | 0.472 |  | 0.035 |

Note: Continuous variables are presented as mean (standard deviation) or median (interquartile range), as appropriate to the analysis. SD, standard deviation; IQR, interquartile range; SBP, Systolic Blood Pressure; DBP, Diastolic Blood Pressure; FBG, Fasting Blood Glucose; HDL, High-Density Lipoprotein; Hct, Hematocrit; WBC, White Blood Cell; RBC, Red Blood Cell; MI, Myocardial Infarction; DM, Diabetes Mellitus; ODC, Obesity Depression Comorbidity; CA, Calcium.

**Supplementary Table 2:** KNHANES baseline information.

| **Variable** | **Test Set (n=713)** | **Training Set (n=1665)** | **Verification**  **Set 1 (n=477)** | **Verification**  **Set 2 (n=502)** | **p-value** | **Test** | **SMD** |
| --- | --- | --- | --- | --- | --- | --- | --- |
| **Age, Mean (SD)** | 45.38 (13.87) | 45.44 (14.29) | 47.48 (14.14) | 46.14 (13.96) | 0.033 |  | 0.083 |
| **Female, n (%)** | 309 (43.3%) | 701 (42.1%) | 186 (39.0%) | 207 (41.2%) | 0.501 | exact | 0.047 |
| **Income level, n (%)** | |  |  |  | 0.292 | exact | 0.096 |
| Low | 155 (21.7%) | 383 (23.0%) | 96 (20.1%) | 87 (17.3%) |  |  |  |
| Medium-Low | 185 (25.9%) | 404 (24.3%) | 116 (24.3%) | 125 (24.9%) |  |  |  |
| Medium-High | 180 (25.2%) | 401 (24.1%) | 130 (27.3%) | 133 (26.5%) |  |  |  |
| High | 193 (27.1%) | 477 (28.6%) | 135 (28.3%) | 157 (31.3%) |  |  |  |
| **Education level, n (%)** | |  |  |  | 0.819 | exact | 0.079 |
| Elementary or less | 103 (15.4) | 336 (16.7) | 58 (17.3) | 46 (13.8) |  |  |  |
| Middle School | 78 (11.6) | 207 (10.3) | 32 (9.5) | 44 (13.2) |  |  |  |
| High School | 269 (40.1) | 807 (40.0) | 135 (40.2) | 134 (40.1) |  |  |  |
| College or Higher | 221 (32.9) | 666 (33.0) | 111 (33.0) | 110 (32.9) |  |  |  |
| **Married, n (%)** | 603 (84.6%) | 1389 (83.4%) | 414 (86.8%) | 408 (81.3%) | 0.111 | exact | 0.081 |
| **SBP, Mean (SD)** | 116.83 (16.05) | 116.44 (15.56) | 117.97 (8.57) | 116.80 (8.00) | 0.219 |  | 0.068 |
| **DBP, Mean (SD)** | 76.34 (10.62) | 75.71 (10.38) | 77.25 (5.06) | 76.73 (4.68) | 0.005 |  | 0.106 |
| **Height, Mean (SD)** | 164.43 (9.23) | 165.14 (9.21) | 165.27 (8.98) | 166.20 (8.75) | 0.011 |  | 0.100 |
| **FBG, Mean (SD)** | 96.72 (17.83) | 97.22 (21.93) | 97.36 (20.18) | 97.95 (19.29) | 0.783 |  | 0.033 |
| **Insulin, Median [IQR]** | 8.81 [6.88, 10.83] | 8.63 [6.74, 11.31] | 9.52 [8.59, 11.03] | 9.49 [8.55, 10.90] | <0.001 | nonnorm | 0.066 |
| **Total Cholesterol, Mean (SD)** | 187.96 (34.67) | 188.28 (35.26) | 190.61 (34.02) | 191.15 (35.69) | 0.237 |  | 0.057 |
| **HDL_C, Mean (SD)** | 48.06 (11.26) | 48.23 (10.79) | 50.74 (14.12) | 51.03 (12.78) | <0.001 |  | 0.155 |
| **Triglycerides, Median [IQR]** | 112.00 [75.00, 160.00] | 106.00 [73.00, 161.00] | 108.00 [74.00, 167.00] | 107.00 [70.00, 161.00] | 0.527 | nonnorm | 0.036 |
| **Hct, Mean (SD)** | 42.14 (4.20) | 42.20 (4.21) | 42.84 (3.87) | 42.90 (4.07) | <0.001 |  | 0.119 |
| **Ferritin, Mean (SD)** | 91.58 (98.58) | 90.21 (89.55) | 100.20 (96.59) | 93.17 (78.54) | 0.207 |  | 0.057 |
| **Serum Creatinine, Mean (SD)** | 0.87 (0.22) | 0.88 (0.18) | 0.87 (0.17) | 0.88 (0.17) | 0.489 |  | 0.050 |
| **WBC count, Mean (SD)** | 8.70 (2.18) | 8.77 (1.84) | 8.68 (1.68) | 8.84 (1.73) | 0.481 |  | 0.050 |
| **RBC count, Mean (SD)** | 4.68 (0.48) | 4.68 (0.47) | 4.69 (0.44) | 4.66 (0.43) | 0.866 |  | 0.028 |
| **Platelet Count, Mean (SD)** | 252.07 (51.28) | 250.33 (51.59) | 250.67 (56.70) | 249.46 (54.00) | 0.841 |  | 0.026 |
| **Vitamin D, Mean (SD)** | 20.07 (6.83) | 19.81 (6.81) | 18.33 (5.45) | 17.80 (5.26) | <0.001 |  | 0.227 |
| **Hypertension, yes** | 118 (16.5%) | 280 (16.8%) | 88 (18.4%) | 75 (14.9%) | 0.534 | exact | 0.048 |
| **Hyperlipidemia,yes** | 59 (8.3%) | 134 (8.0%) | 49 (10.3%) | 48 (9.6%) | 0.379 | exact | 0.046 |
| **Stroke, yes** | 7 (1.0%) | 21 (1.3%) | 6 (1.3%) | 3 (0.6%) | 0.663 | exact | 0.039 |
| **MI, yes** | 15 (2.1%) | 28 (1.7%) | 12 (2.5%) | 10 (2.0%) | 0.624 | exact | 0.030 |
| **Arthritis, yes** | 144 (20.2%) | 332 (19.9%) | 97 (20.3%) | 101 (20.1%) | 0.998 | exact | 0.005 |
| **DM, yes** | 38 (5.3%) | 113 (6.8%) | 25 (5.2%) | 19 (3.8%) | 0.064 | exact | 0.068 |
| **Depression, yes** | 68 (9.5%) | 195 (11.7%) | 51 (10.7%) | 61 (12.2%) | 0.387 | exact | 0.047 |
| **ODC, yes** | 26 (3.6%) | 73 (4.4%) | 19 (4.0%) | 20 (4.0%) | 0.884 | exact | 0.019 |
| **Smoking** |  |  |  |  | 0.96 | exact | 0.051 |
| < 5 packs | 21 (2.9%) | 47 (2.8%) | 15 (3.1%) | 18 (3.6%) |  |  |  |
| ≥ 5 packs | 347 (48.7%) | 829 (49.8%) | 239 (50.1%) | 242 (48.2%) |  |  |  |
| Never | 345 (48.4%) | 789 (47.4%) | 223 (46.8%) | 242 (48.2%) |  |  |  |
| **Drinker, yes** | 644 (90.3%) | 1506 (90.5%) | 447 (93.7%) | 467 (93.0%) | 0.049 | exact | 0.078 |
| **Obesity (yes), n (%)** | 285 (40.0%) | 642 (38.6%) | 187 (39.2%) | 176 (35.1%) | 0.352 | exact | 0.053 |
| **Obesity Type Code, n (%)** |  |  |  |  | 0.628 | exact | 0.085 |
| Non-obese | 428 (60.0%) | 1023 (61.4%) | 290 (60.8%) | 326 (64.9%) |  |  |  |
| Abdominal Obesity Only | 22 (3.1%) | 55 (3.3%) | 16 (3.4%) | 15 (3.0%) |  |  |  |
| General Obesity Only | 128 (18.0%) | 299 (18.0%) | 80 (16.8%) | 91 (18.1%) |  |  |  |
| Combined Obesity | 135 (18.9%) | 288 (17.3%) | 91 (19.1%) | 70 (13.9%) |  |  |  |
| **Food, Mean (SD)** | 33.23 (22.95) | 32.73 (20.11) | 31.05 (19.83) | 31.27 (19.80) | 0.165 |  | 0.064 |
| **Energy, Mean (SD)** | 44.65 (27.28) | 44.41 (25.13) | 43.31 (26.75) | 40.87 (25.16) | 0.040 |  | 0.080 |
| **Water, Mean (SD)** | 22.69 (18.03) | 22.31 (15.85) | 21.05 (15.95) | 21.81 (15.88) | 0.345 |  | 0.055 |
| **Protein, Mean (SD)** | 1.57 (0.95) | 1.60 (0.94) | 1.58 (1.04) | 1.54 (1.11) | 0.667 |  | 0.030 |
| **Fat, Mean (SD)** | 0.86 (0.97) | 0.91 (0.84) | 0.92 (0.90) | 0.94 (1.27) | 0.480 |  | 0.042 |
| **Carbohydrate, Mean (SD)** | 7.30 (4.92) | 7.11 (4.31) | 6.62 (3.96) | 6.24 (3.20) | <0.001 |  | 0.150 |
| **CA, Mean (SD)** | 11.34 (8.39) | 11.61 (8.81) | 10.65 (8.67) | 10.91 (8.60) | 0.120 |  | 0.064 |
| **Phosphorus, Mean (SD)** | 26.47 (14.16) | 26.71 (14.09) | 25.20 (13.51) | 24.57 (13.93) | 0.009 |  | 0.092 |
| **Fe, Mean (SD)** | 0.33 (0.32) | 0.34 (0.29) | 0.31 (0.21) | 0.32 (0.26) | 0.251 |  | 0.056 |
| **Na, Mean (SD)** | 111.22 (72.93) | 112.80 (69.86) | 104.53 (70.04) | 98.97 (68.22) | 0.001 |  | 0.115 |
| **K, Mean (SD)** | 71.24 (43.81) | 70.41 (47.10) | 63.87 (31.17) | 63.66 (35.09) | <0.001 |  | 0.123 |
| **Vitamin A, Mean (SD)** | 19.29 (29.65) | 19.07 (26.86) | 16.43 (14.10) | 18.56 (22.90) | 0.210 |  | 0.069 |
| **Carotene, Mean (SD)** | 97.75 (158.27) | 99.98 (156.50) | 82.75 (73.11) | 97.59 (134.46) | 0.149 |  | 0.072 |
| **Retinol, Mean (SD)** | 2.47 (8.93) | 2.38 (6.06) | 2.19 (3.09) | 2.40 (3.60) | 0.894 |  | 0.028 |
| **Thiamine, Mean (SD)** | 0.03 (0.03) | 0.03 (0.02) | 0.03 (0.02) | 0.03 (0.03) | 0.762 |  | 0.021 |
| **Riboflavin, Mean (SD)** | 0.03 (0.02) | 0.03 (0.02) | 0.03 (0.03) | 0.03 (0.02) | 0.785 |  | 0.028 |
| **Niacin, Mean (SD)** | 0.37 (0.23) | 0.38 (0.24) | 0.37 (0.26) | 0.37 (0.26) | 0.794 |  | 0.024 |
| **Vitamin C, Mean (SD)** | 2.65 (3.20) | 2.48 (2.51) | 2.23 (1.63) | 2.42 (3.37) | 0.067 |  | 0.084 |
| **Region, n (%)** |  |  |  |  | 0.85 | exact | 0.03 |
| *Seoul* | 202 (28.3%) | 470 (28.2%) | 135 (28.3%) | 142 (28.3%) |  |  |  |
| *Busan* | 43 (6.0%) | 102 (6.1%) | 29 (6.1%) | 31 (6.2%) |  |  |  |
| *Daegu* | 45 (6.3%) | 106 (6.4%) | 30 (6.3%) | 32 (6.4%) |  |  |  |
| *Incheon* | 66 (9.3%) | 152 (9.1%) | 44 (9.2%) | 46 (9.2%) |  |  |  |
| *Gwangju* | 31 (4.3%) | 70 (4.2%) | 20 (4.2%) | 21 (4.2%) |  |  |  |
| *Daejeon* | 32 (4.5%) | 73 (4.4%) | 21 (4.4%) | 22 (4.4%) |  |  |  |
| *Ulsan* | 23 (3.2%) | 54 (3.2%) | 15 (3.1%) | 16 (3.2%) |  |  |  |
| *Sejong* | 219 (30.7%) | 512 (30.7%) | 146 (30.6%) | 154 (30.7%) |  |  |  |
| *Gyeonggi* | 35 (4.9%) | 82 (4.9%) | 23 (4.8%) | 25 (5.0%) |  |  |  |
| *Gangwon* | 41 (5.8%) | 98 (5.9%) | 28 (5.9%) | 29 (5.8%) |  |  |  |
| *Chungbuk* | 45 (6.3%) | 107 (6.4%) | 31 (6.5%) | 32 (6.4%) |  |  |  |
| *Chungnam* | 43 (6.0%) | 101 (6.1%) | 29 (6.1%) | 31 (6.2%) |  |  |  |
| *Jeonbuk* | 49 (6.9%) | 115 (6.9%) | 33 (6.9%) | 35 (7.0%) |  |  |  |
| *Jeonnam* | 50 (7.0%) | 116 (7.0%) | 33 (6.9%) | 35 (7.0%) |  |  |  |
| *Gyeongbuk* | 53 (7.4%) | 124 (7.4%) | 35 (7.3%) | 37 (7.4%) |  |  |  |
| *Gyeongnam* | 25 (3.5%) | 59 (3.5%) | 17 (3.6%) | 18 (3.6%) |  |  |  |
| *Jeju* | 2 (0.3%) | 12 (0.7%) | 3 (0.6%) | 3 (0.6%) |  |  |  |

Note: Continuous variables are presented as mean (standard deviation) or median (interquartile range), as appropriate to the analysis. SD, standard deviation; IQR, interquartile range; SBP, Systolic Blood Pressure; DBP, Diastolic Blood Pressure; FBG, Fasting Blood Glucose; HDL, High-Density Lipoprotein; Hct, Hematocrit; WBC, White Blood Cell; RBC, Red Blood Cell; MI, Myocardial Infarction; DM, Diabetes Mellitus; ODC, Obesity Depression Comorbidity; CA, Calcium.

**Supplementary Table 3.** Model Performance Ranking (Based on Brier Score)

| Rank | Model | Brier Score |
| --- | --- | --- |
| 1 | XGBoost | 0.0240 |
| 2 | Random Forest | 0.0244 |
| 3 | K-Nearest Neighbors | 0.0513 |
| 4 | SVM (RBF) | 0.0694 |
| 5 | Decision Tree | 0.0853 |
| 6 | Naive Bayes | 0.1083 |
| 7 | Logistic Regression | 0.1175 |

The Brier Score measures the accuracy of probabilistic predictions. It ranges from 0 to 1, where a lower score indicates better performance.

**Supplementary Table 4**. Model Performance Metrics on the Independent Test Set

| **Metric** | **Value** |
| --- | --- |
| Accuracy | 0.7293 |
| Precision | 0.0846 |
| Recall (Sensitivity) | 0.6538 |
| F1-Score | 0.1498 |
| AUC | 0.7500 |

**Supplementary Table 5**. Confusion Matrix on the Test Set

|  | **Predicted: Negative** | **Predicted: Positive** |
| --- | --- | --- |
| **Actual: Negative** | 503 (True Negative) | 184 (False Positive) |
| **Actual: Positive** | 9 (False Negative) | 17 (True Positive) |

**Supplementary Table 6**. Optimal Model Training Parameters

| **Parameter** | **Value** | **Description** |
| --- | --- | --- |
| Best Iteration | 44 | The number of boosting rounds at which the model performed best on the validation set. |
| Best Validation AUC | 0.7500 | The highest Area Under the ROC Curve achieved on the hold-out validation set during training. |
| Early Stopping Round | 50 | Training was halted after 50 rounds without improvement in validation AUC. |
| Applied Decision Threshold | 0.1 | The probability cutoff used to assign class labels (Probability > 0.1 = Class 1). |

**Supplementary Table 7**. Performance Differences Between Internal Validation Queues

| Metric | 2011 Validation | 2012 Validation | Difference (Δ) |
| --- | --- | --- | --- |
| AUC | 0.783 | 0.744 | +0.039 |
| Sensitivity | 0.842 | 0.700 | +0.142 |
| Specificity | 0.594 | 0.627 | -0.033 |
| Precision | 0.079 | 0.072 | +0.007 |
| F1-Score | 0.145 | 0.131 | +0.014 |
| Accuracy | 0.604 | 0.629 | -0.026 |

**Supplementary Table 8:** External Validation of Model Performance Differences in NHANES

| Model | AUC | Accuracy | Precision | Recall | F1 Score |
| --- | --- | --- | --- | --- | --- |
| XGBoost | 0.8954 | 0.9420 | 0.9763 | 0.9611 | 0.9687 |
| Random Forest | 0.8580 | 0.9758 | 0.9747 | 0.9851 | 0.9872 |
| SVM (RBF) | 0.8311 | 0.9469 | 0.9461 | 0.9546 | 0.9723 |
| K-Nearest Neighbors | 0.7949 | 0.9300 | 0.9386 | 0.9896 | 0.9634 |
| Logistic Regression | 0.7776 | 0.9300 | 0.9343 | 0.9948 | 0.9636 |
| Naive Bayes | 0.7590 | 0.8961 | 0.9409 | 0.9482 | 0.9445 |
| Decision Tree | 0.6666 | 0.9372 | 0.9478 | 0.9870 | 0.9670 |


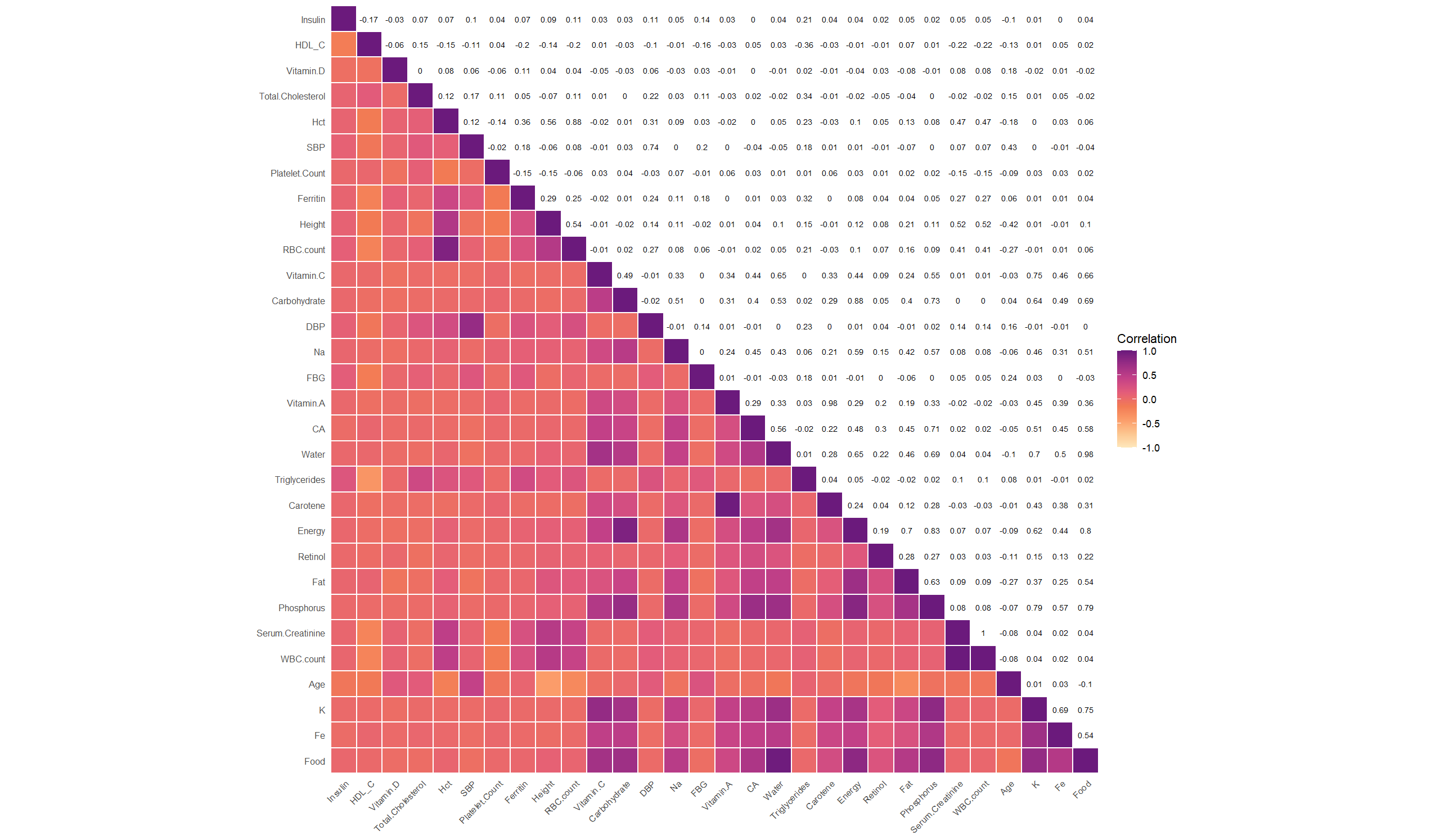


**Supplementary Figure 1:** Pearson association coefficient plot (prior to removing collinear variables)

**
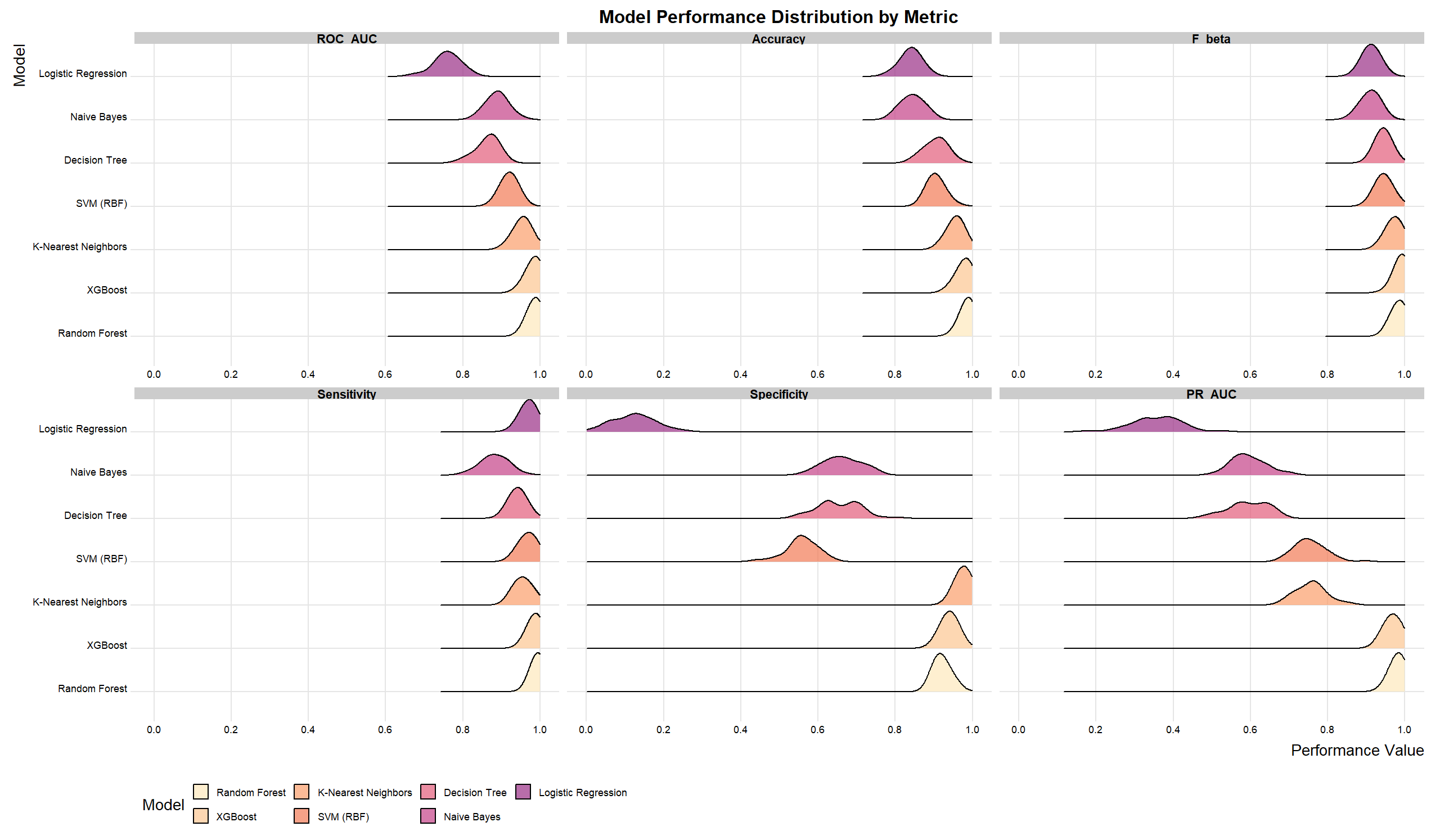
**

**Supplementary Figure 2:** Ridge Plot Distribution of Performance Metrics for Seven Machine Learning Models

X-axis: AUC values; Y-axis: Model types; Curve height indicates probability density of ten-fold cross-validation performance distribution.


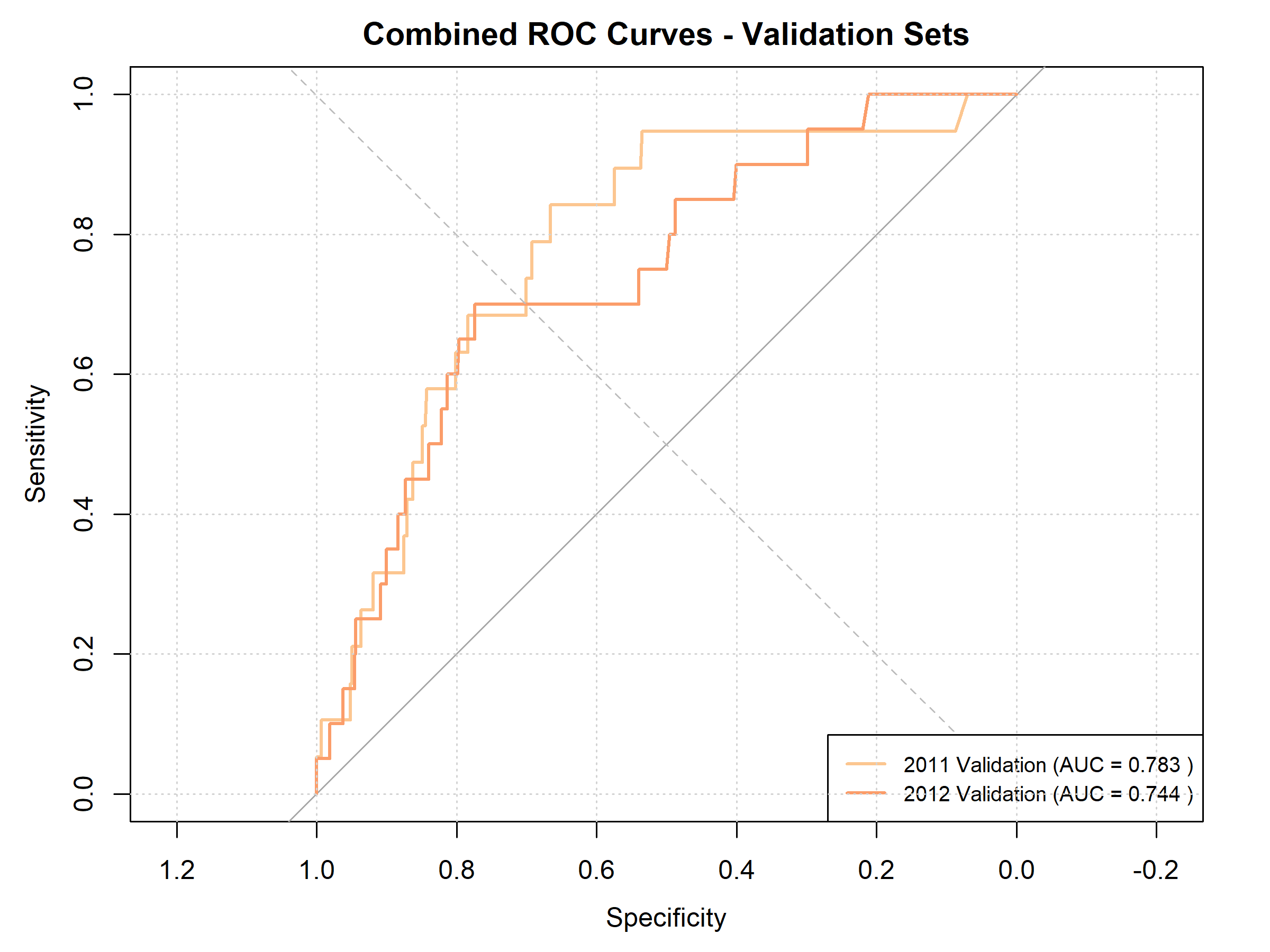


**Supplementary Figure 3:** ROC curve for the KNHANES internal validation set


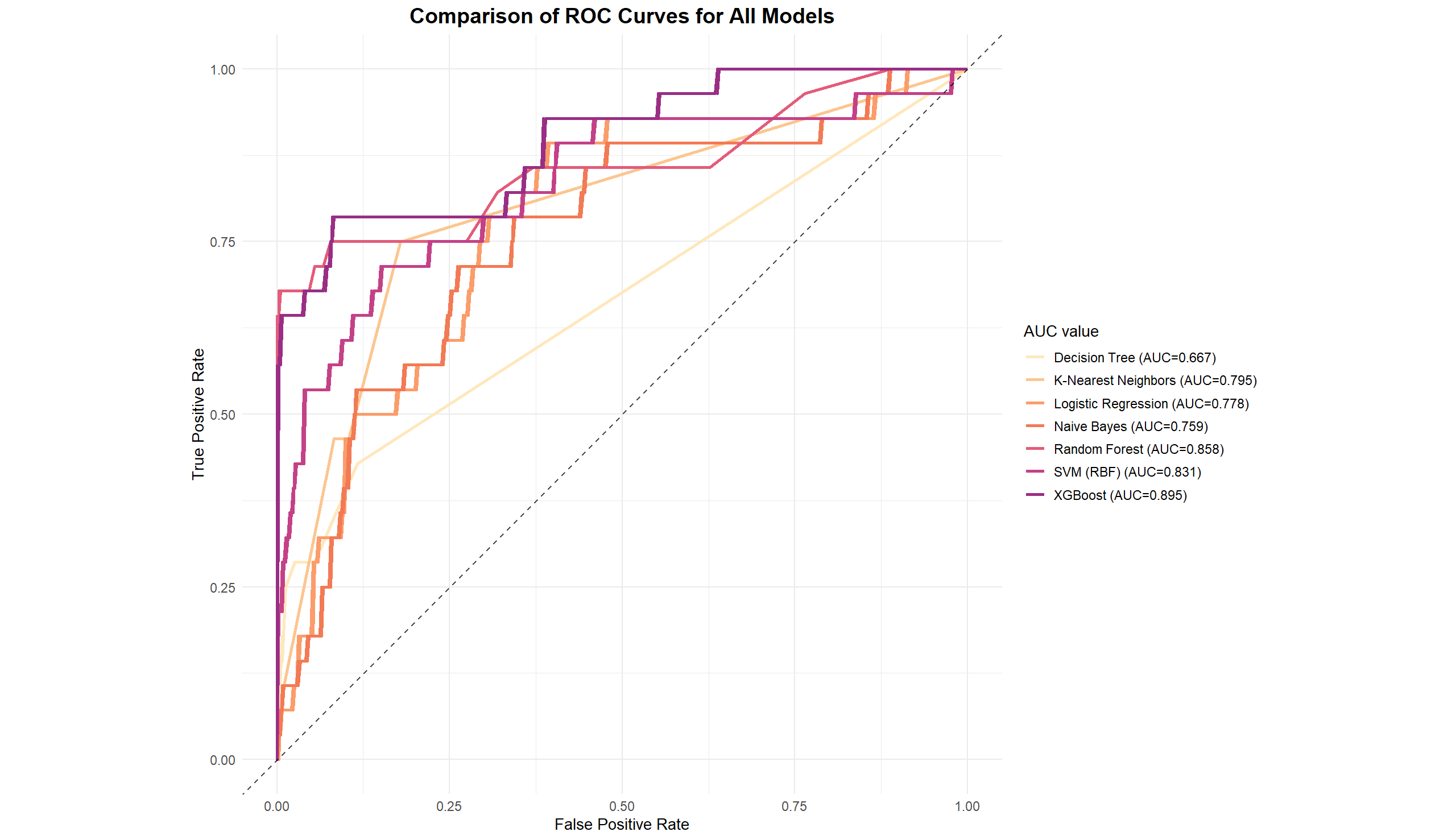


**Supplementary Figure 4:** ROC curve for the NHANES external validation set


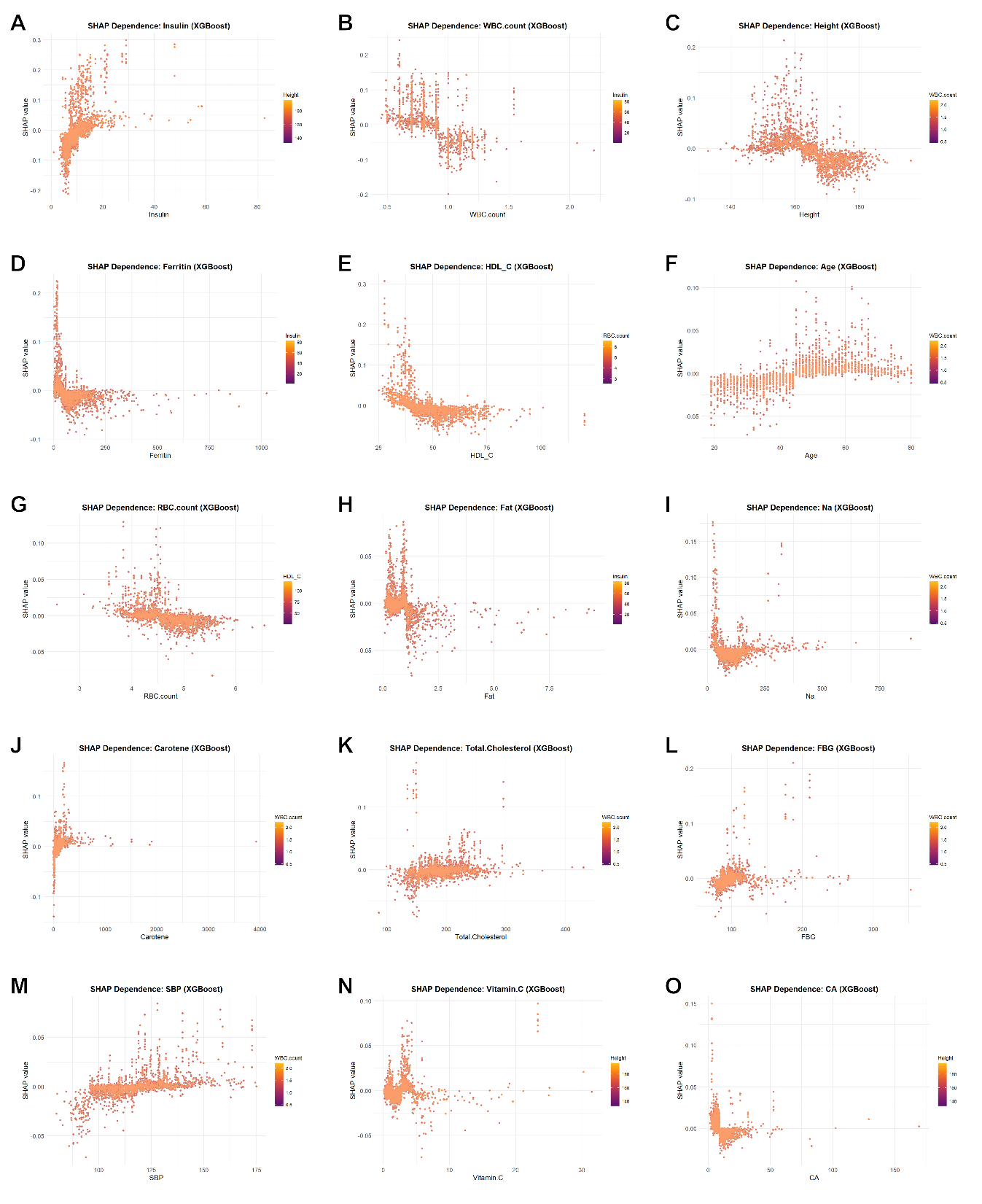


**Supplementary Figure 5:** SHAP Dependency Plot for KNHANES Data.

(A-O) Variables: Insulin, White Blood Cell Count (WBC count), Height, Ferritin, High-Density Lipoprotein Cholesterol (HDL_C), Age, Red Blood Cell Count (RBC count), Fat Intake, Sodium Intake, β-Carotene, Total Cholesterol, Fasting Blood Glucose (FBG), Systolic Blood Pressure (SBP), Vitamin C Intake, and Calcium Intake (CA). X-axis: Actual feature measurements; Y-axis: SHAP values.


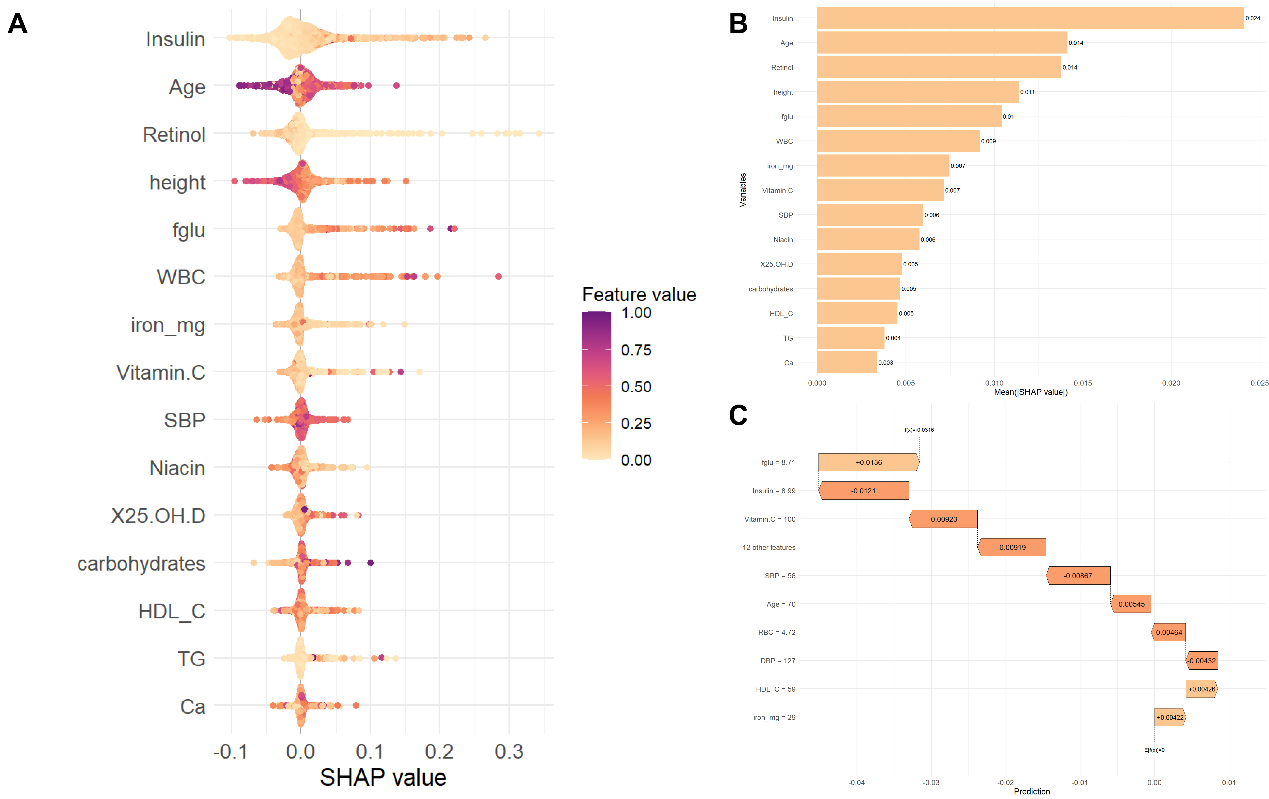


**Supplementary Figure 6:** SHAP Analysis of XGBoost Model on NHANES Cohort

(A) SHAP beeswarm plot. (B) SHAP global importance bar plot. (C) SHAP waterfall plot


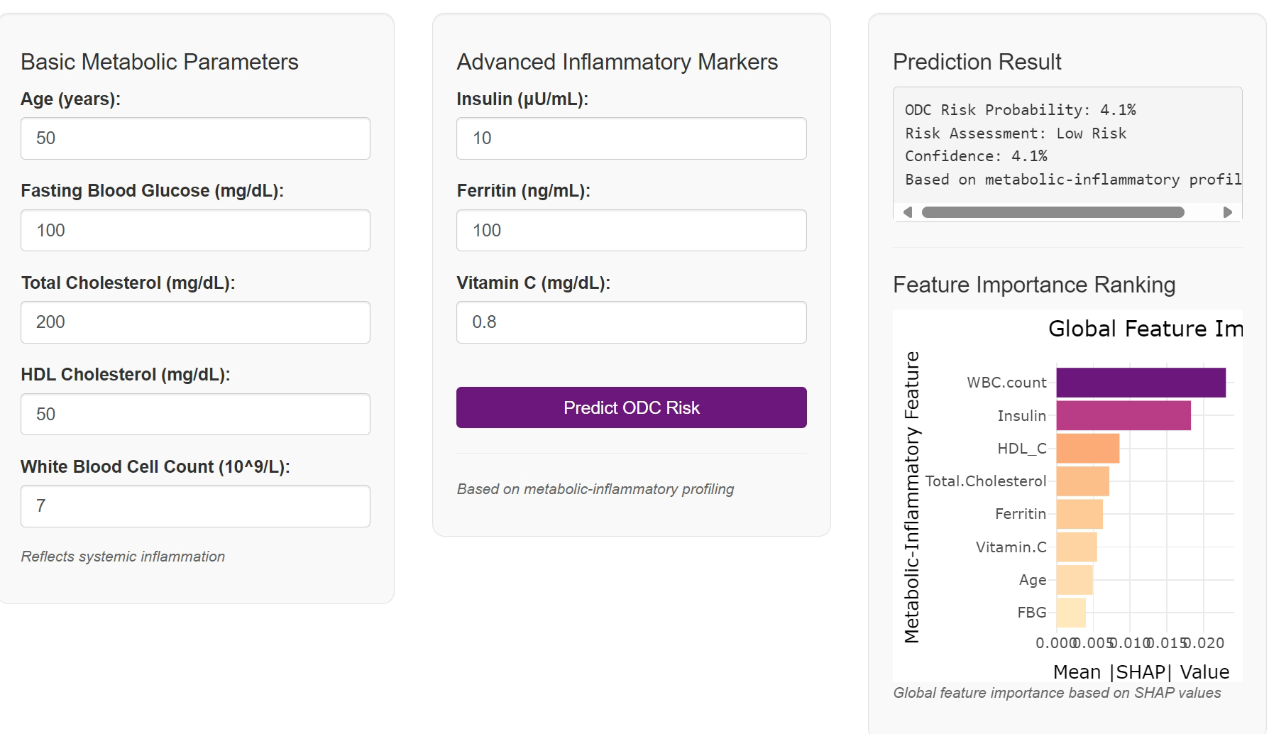


**Supplementary Figure 7: Real-time Visualization of Risk Probability Stratification Output from Online Prediction Tool**
